# Braun on Eclampsia during Pregnancy

**Published:** 1854-11

**Authors:** 


*Braun on Eclampsia during Pregnancy*-—This author has recently published a lengthy paper on the above interesting subject, of which the following is a very brief abstract:

1st. Convulsions may arise, during this period, from hysteria, epilepsy, cerebral diseases, poisons, or uraemia,—resulting from Bright's disease.

2d. Their most common causes are uraemia and Bright's disease.

3d. The least frequent causes are primary cerebral diseases; and when these occur in connection with albuminous nephritis, they are the *results*, not the causes, of the convulsions.

4th. Hysteria and epilepsy may exist, during gestation, in a chronic form, so as to exercise no injurious influence either on pregnancy or labor, or on the life of the child; and they may be quite unconnected with Bright's disease.

5th. Convulsions in every form may occur from these causes in the unimpregnated female; and also in men, from all of them, except hysteria.

6th. The altered constitution of the blood, and the detention of the venous blood in the kidneys by the pressure of the enlarged uterus, are most commonly the causes of the Morbus Brightii, which occurs during pregnancy.

7th. Convulsions are caused by the urea, which is uneliminated from the blood on account of the renal disease, becoming changed into the carbonate of ammonia.

8th. When, in cases of Bright's disease during pregnancy, we find carbonate of ammonia in the blood, we may prognosticate the occurrence of convulsions; but when, at this time, the urea in the blood exists merely in small quantities, or chemically unchanged, we need not dread eclampsia.

9th. Parturition and uterine irritation neither cause this chemical transformation, nor occasion uraemic eclampsia.

10th. The abortions which so frequently happen during uraemic convulsions, are the results, not the causes of the eclampsia.

11th. There is no connection between eclampsia and labor-pains.

12th. Albuminuria does not result from convulsions arising from functional interruptions, and does not generally occur in those of an epileptical and hysterical character.

13th. Albuminuria continues throughout pregnancy, although the eclampsia attacks may have ceased; but when convulsions cease *after labor*, it soon disappears, provided the renal disease be only in the incipient stage.

14th. The disappearance of the albuminuria after parturition, is principally due to the diminished volume of the uterus.

15th. Morbus Brightii (without convulsion) may be palliated, but not removed, during pregnancy, although it readily yields to remedies after parturition.

16th. Albuminuria occurs in all cases of eclampsia which do not depend upon hysteria, epilepsy, primary cerebral diseases, or poisons,

17th. Epileptic convulsions may occur simultaneously with those from Bright's disease and uraemia.

18th. Uraemic convulsions, when frequently recurring, occasion the death of the fœtus; but its life is not endangered by those from hysteria or epilepsy.

19th. During an attack of uraemic eclampsia, reflex sensibility is almost wholly suspended; and after it we oftener find ædema and anæmia of the brain, than hyperæmia and consecutive apoplexy before it.

20th. Venesection (according to Kiwisch, Litzman, Sedgwick, Blot, and King) is injurious in eclampsia; and the author has found its action very uncertain in uraemia. He considers inhalations of chloroform to be the best and safest means we possess for subduing and removing uraemic convulsions, both during pregnancy and after labor.

21st. The most certain diuretics for removing the uraemia of Bright's disease are the benzoic, tartaric and citric acids.

22d. The artificial induction of premature labor diminishes the danger of uraemic eclampsia alike to the mother and child; but this practice should not be universally resorted to in cases of Bright's disease during pregnancy, but only when necessitated by the occurrence of convulsions. Braun considers the tampon as the best method for its induction when necessary.

[The Editor of the Monthly Journal remarks: The treatment here proposed appears to us by no means universally applicable. Where the convulsions occur during pregnancy, or in an anaemic patient, or where, as happens in infantile eclampsia, they depend rather upon a superpolarity of the cerebro-spinal system that on actual toxaemia, we consider the inhalation of chloroform as unquestionably the best treatment for arresting the paroxysms. But when, on the other hand, they occur during parturition, or in the puerperal state, in a robust, plethoric patient, we should be inclined, from all we have seen of the disease, to place more confidence in copious venesection, smart purgatives, and cold to the head.

Moreover, for the induction of premature labor in such cases, we should be inclined to prefer the use of sponge-tents and uterine douches to the clumsier method of the tampon.]—*Edinburgh Journ. Med. Science.*

